# Determinants of Schizophrenia Endophenotypes Based on Neuroimaging and Biochemical Parameters

**DOI:** 10.3390/biomedicines9040372

**Published:** 2021-04-01

**Authors:** Amira Bryll, Wirginia Krzyściak, Paulina Karcz, Maciej Pilecki, Natalia Śmierciak, Marta Szwajca, Anna Skalniak, Tadeusz J. Popiela

**Affiliations:** 1 Department of Radiology, Jagiellonian University Medical College, 31-501 Krakow, Poland; amira.bryll@uj.edu.pl; 2 Department of Medical Diagnostics, Jagiellonian University Medical College, 30-688 Krakow, Poland; 3 Department of Electroradiology, Jagiellonian University Medical College, 31-126 Krakow, Poland; paulina.karcz@uj.edu.pl; 4 Department of Child and Adolescent Psychiatry, Faculty of Medicine, Jagiellonian University Medical College, 31-501 Krakow, Poland; maciej.pilecki@uj.edu.pl (M.P.); natalia.smierciak@uj.edu.pl (N.Ś.); marta.szwajca@uj.edu.pl (M.S.); 5 Department of Endocrinology, Faculty of Medicine, Jagiellonian University Medical College, 31-501 Krakow, Poland; anna.skalniak@uj.edu.pl

**Keywords:** schizophrenia, endophenotypes, magnetic resonance spectroscopy, glutamatergic metabolites, anterior cingulate gyrus

## Abstract

Despite extensive research, there is no convincing evidence of a reliable diagnostic biomarker for schizophrenia beyond clinical observation. Disorders of glutamatergic neurotransmission associated with N-methyl-D-aspartate (NMDA) receptor insufficiency, neuroinflammation, and redox dysregulation are the principal common mechanism linking changes in the periphery with the brain, ultimately contributing to the emergence of negative symptoms of schizophrenia that underlie differential diagnosis. The aim of the study was to evaluate the influence of these systems via peripheral and cerebral biochemical indices in relation to the patient’s clinical condition. Using neuroimaging diagnostics, we were able to define endophenotypes of schizophrenia based on objective laboratory data that form the basis of a personalized approach to diagnosis and treatment. The two distinguished endophenotypes differed in terms of the quality of life, specific schizophrenia symptoms, and glutamatergic neurotransmission metabolites in the anterior cingulate gyrus. Our results, as well as further studies of the excitatory or inhibitory balance of microcircuits, relating the redox systems on the periphery with the distant regions of the brain might allow for predicting potential biomarkers of neuropsychiatric diseases, including schizophrenia. To the best of our knowledge, our study is the first to identify an objective molecular biomarker of schizophrenia outcome.

## 1. Introduction

Schizophrenia is a severe mental disorder, the diagnosis of which is currently based solely on clinical criteria [1]. There is a lack of laboratory biomarkers related to the etiopathogenesis of this disease. A biomarker is ‘an objectively measured trait that reflects a normal biological or pathological process, or response to a pharmacological intervention’ [2]. Thus, it is a disease-specific indicator of the presence or intensity of a biological process directly related to the patient’s clinical symptoms or the effects of a certain disorder [3].

Despite scientific progress in understanding the pathophysiological basis of schizophrenia, many potential emerging predictors do not meet the biomarker criteria. However, due to rapidly developing neuroimaging techniques, there is growing hope that those techniques might provide biomarkers in the future, which would correlate with the clinical outcome of the disease and predict the patients’ endophenotypes [4].

Due to the complexity of the clinical picture of schizophrenia, the concept of endophenotypes is increasingly used in research on this disorder. An endophenotype is a quantitative measure based on neurobiological data that is created on the basis of laboratory measurements, which is not an estimate from clinical examination [5,6]. The clinical picture of schizophrenia might include classic subtypes [7] as well as a division, depending on the dominant component of negative and positive symptoms [8]. In addition, according to the latest International Classification of Diseases 11th classification, there is a division based on the stage of the illness (first episode/multiple episodes/solid course) and the severity of symptoms (currently symptomatic/partial remission/full remission) [8]. The boundaries between schizophrenia and schizoaffective and affective disorder with psychotic symptoms are also blurred. Schizophrenia might also manifest in a form with an expressed obsessive-compulsive component, obsessive-compulsive symptoms might be an initial stage in the development of schizophrenia, or both disorders could be comorbid [9]. The literature on the subject also includes descriptions of personality traits of schizotypes that predispose one to the development of schizophrenia or with a clinical picture similar to schizophrenia (schizotypal personality) [10]. As reported in the literature on the subject, this definition, as well as the progress made in research on the human genome and neuroimaging techniques in psychiatry, shows the polygenic nature of schizophrenia with its various causative factors, i.e., genetic, immunological, biochemical, and environmental [7,8,9,10,11,12,13,14,15].

Understanding the classic definition of endophenotypes and the Research Domains Criteria announced by the National Institute of Mental Health [16,17] allows us to assume that the matter concerning the boundaries of division is still open. As they emphasize, however, there are no data supporting this theory (only theoretical statements) [18,19], hence, the theory itself is purely an empirical question that should be solved by statistical methods designed to explain an existing scientific problem.

The starting point of the presented work was the hidden structure of endophenotypes underlying the classical understanding of this term. This gives an opportunity to build a model based on empirical laboratory data from neuroimaging and a number of other laboratory variables that connect the concept of pathology of schizophrenia with subtypes, thus outlining a new perspective on the classical understanding of its diagnosis.

Different studies suggest that several key brain regions involved in the pathophysiology of schizophrenia, such as the frontal cortex, dorsal anterior cingulate cortex (ACC), putamen, and temporal pole, share metabolic abnormalities or altered perfusion in patients with schizophrenia [20].

The progression of the disease also seems to be an important issue in this context. On the one hand, it is associated with the period of untreated psychosis, as we showed in our previous studies [21]. On the other hand, it might be related to the functioning of the ACC itself, which is seen especially in patients taking antipsychotic drugs who show greater ACC activation with associated better performance. It is, however, unclear whether this is directly caused by the influence of drugs, or by a reduction of symptoms [22]. Atrophy of the cerebral cortex, mainly related to the limited functional connectivity of the ACC, correlates with the most unpleasant predictors of disability in schizophrenia that do not respond to the classic form of treatment, i.e., negative symptoms. It also correlates with cognitive symptoms of schizophrenia but has no relation with the severity of positive symptoms [23,24]. Studies determining the concentration of metabolites proposed ACC as a potential source of biomarkers related to the risk of psychosis in early adolescence [25]. These data provide evidence of association between glutamatergic transmission metabolites and symptomatology, which might predict the development of psychosis in the future [26]. Thus, assessment of brain metabolites might thus play an important role as an early biomarker of schizophrenia risk, especially among young relatives of people with schizophrenia [27].

Therefore, the use of tools such as proton magnetic resonance spectroscopy (1H-MRS) might likely facilitate the identification of early predictors of schizophrenia, especially in the stage of juvenile psychosis, when the brain develops specific features related to changes in metabolite concentration, in particular reflecting changes in glutamatergic transmission [24].

Glutamate (also known as glutamic acid) is synthesized from glutamine by glutaminase in the central nervous system, and belongs to one of the main excitatory neurotransmitters changing neuronal activity and synaptic functions in the brain [28]. However, excessive glutamate release from presynaptic axon terminals can also cause neuronal death and permanent brain damage in a process known as excitotoxicity [29]. This phenomenon is associated with the pathogenesis of many debilitating human neurological diseases, such as stroke, amyotrophic lateral sclerosis, and epilepsy [30].

**Figure 1:**
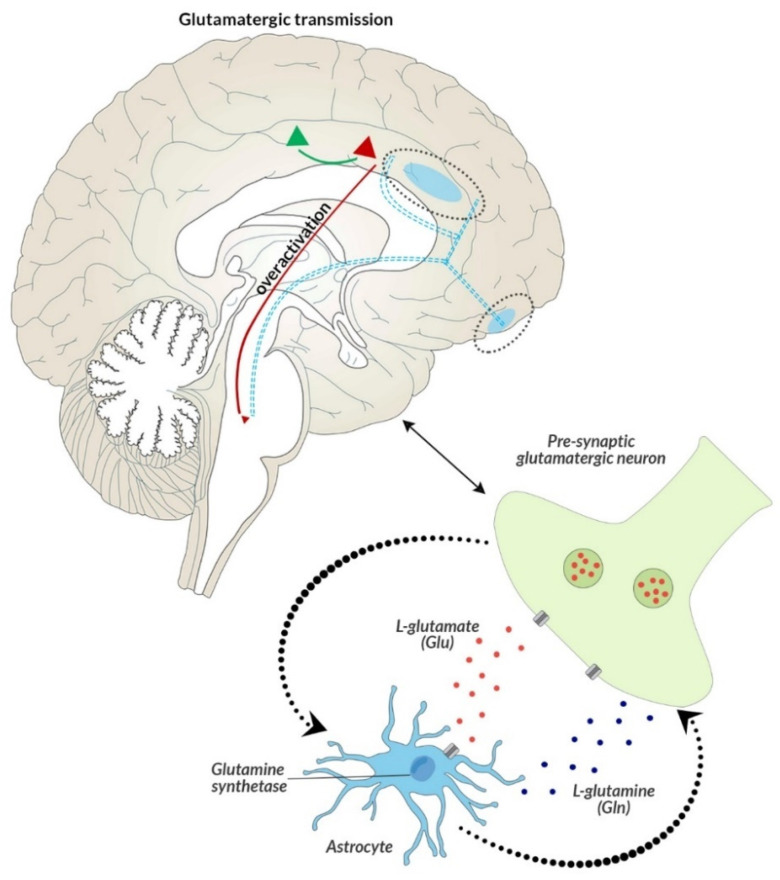
Potential mechanism explaining the occurrence of negative symptoms, and the developing resistance to classical neuroleptics due to the glutamatergic hypothesis and the NMDA receptor hypofunction. In addition to the glutamate projection pathway in the cortical brain stem, which is associated with the primary GLU neuron, the GABA interneuron, and the secondary GLU neuron, additional synapses in the midbrain with another GABA interneuron that targets neuronal DA projections that return to the frontal cortex, are involved in the projection of negative symptoms in schizophrenia. During normal psychiatric functioning, this circuit is balanced and sufficient DA reaches the frontal cortex without negative symptoms. However, when the pathway is modified with the formation of an additional GABA interneuron (originally: GLU-GABA-GLU-D; after modification: GLU-GABA-GLU-GABA-DA), this change might be associated with the appearance of negative symptoms. In the case of generating positive symptoms, glutamate from the primary GLU neuron reaches hypofunctional NMDA receptors located on the primary GABA interneuron, whose tone disappears in the absence of GLU stimulation and the secondary GLU neuron becomes hyperactive again. In contrast to the mechanism associated with the generation of positive symptoms, the main issue concerns the secondary GLU neuron, which hits another GABA interneuron, which, through a higher than normal GLU signal, releases much higher concentrations of GABA. This results in the inhibition of the final pathway of DA neurons derived from the midbrain and their lower release. This mesocortical DA pathway is now active and does not provide adequate amounts of DA in the frontal cortex, which causes hypofrontality and negative symptoms.

The concentration of glutamatergic metabolites determines higher or lower inhibitory connectivity in ACC and in the anterior part of the insula, shows a negative correlation with the inhibitory effect on excitatory neurons in ACC, and a negative correlation with the severity of social withdrawal in people with first-episode psychosis, as compared to healthy subjects [31]. Glutamate acts mainly postsynaptically by combining with ionotropic receptors (non-NMDA and NMDA), which leads, inter alia, to the depolarization of the cell membrane, and removal of Mg^2+^ ions with simultaneous penetration of Ca^2+^. This consequently contributes to the decarboxylation of glutamic acid and formation of a neurotransmitter with an inhibitory effect, i.e., γ-aminobutyric acid (GABA) (Figure 1). The imbalance between glutamate and brain GABA levels might be a marker of transition to psychosis in high-risk individuals [32]. The knowledge of GABA and glutamate levels at different stages of life of people at risk of developing psychosis might allow better assessment of the neurochemical phenomena underlying mental disorders [33].

The glutamatergic theory of psychosis (related to glutamatergic transmission metabolites), induced by ketamine or other NMDA receptor antagonists, allowed for the distinction between the classic psychosis of schizophrenia in a number of studies. It is mainly associated with positive symptoms, and the form of this disease is associated with additional negative and cognitive symptoms [34,35], which were not taken into account in the treatment of the commonly understood form of the disease [36], described in the early 1980s [37].

The mechanism of the observed changes might be concentrated on the increased ratio of glutamine to glutamate that is associated with an increased flow of excitatory neurotransmitters, and thus with glutamatergic hyperactivity or defective neural-glial coupling [38]. Abnormalities in glutamatergic neurotransmission are associated in this case with the function of glial cells in the prefrontal cortex (PFC) [39], abnormal volume of the brain ventricles [40], and abnormalities in the level of synaptic proteins [41]. These discoveries became the starting point of the latest pharmacological strategies based on NMDA antagonist models, to restore cognitive functions and reduce negative symptoms in schizophrenia [42]. However, cortical-limbic hyperactivity is induced by the administration of NMDA receptor antagonists, initiating the behavioral disturbances observed in schizophrenia, which is explained by the loss of glutamatergic function with excessive dopaminergic activity. Imbalance of the glutaminergic and dopaminergic systems in different regions of the brain might result in anti-kinetic effects or the development of psychosis [43]. The precise determination of the glutamatergic function related to Glu and Gln levels seems to be a limitation of the reported contradictory theories, due to signal-to-noise overlap and limitations of spectral resolution in magnetic resonance spectroscopy (MRS), therefore, in many works, the amounts of Glu–Gln are shown together as Glx [44]. For this reason, there might be misinterpretations of the observed changes, e.g., some studies report a decrease in the Glx levels during acute episodes of psychosis, while in others, the observed relationships are quite different. The physiological significance of the Glx results remains unclear, and MRS techniques distinguishing between the levels of Glu and Gln in different regions of the human brain could provide a solution to the existing problem and an important advance in the diagnosis of these debilitating diseases.

According to some of the research, glutamatergic-mediated excitotoxicity might be one of the mechanisms underlying resistance to classical neuroleptics seen in drug-resistant schizophrenia. The mentioned excitotoxicity associated with increased glutamatergic transmission metabolites levels would explain the weakened differentiation of oligodendrocytes with a higher sensitivity of the latter to inflammation and oxidative stress [45].

The most obvious evidence that oxidative stress might have an influence on the development of schizophrenia is prenatal exposure to hunger, which results in altered hippocampal morphology and impaired memory in the offspring, i.e., abnormalities consistent with the pathophysiology of schizophrenia [46].

Oxidative stress can be both a cause, an effect, and an attempt to combat pathological processes in the body [47]. The latter turned out to be particularly important in the context of parameters that form the so-called microcircuits, i.e., total cholesterol, triglycerides (TG), low-density lipoprotein cholesterol (LDL), high-density lipoprotein cholesterol (HDL), cortisol, and sodium ions, which play a significant role in the initiation of inflammation and assessment of cardiovascular risk in schizophrenics [48,49,50].

The role of oxidative stress related to oxidant–antioxidant imbalance in the pathophysiology of schizophrenia is emphasized by studies assessing the level of these indicators in the blood and peripheral tissues with clear differences between people with schizophrenia and healthy controls [51,52]. Nevertheless, it is unclear how biochemical changes in the periphery are related to neurochemical changes in the brain. There are few studies that point out a positive correlation between glutathione (which is the main intracellular non-enzymatic antioxidant) in plasma, with the levels of glutathione-dependent metabolites associated with glutamatergic transmission in the brains of schizophrenics, as compared to healthy controls [53]. The levels of these metabolites positively correlate with the neuropsychological assessment in patients with schizophrenia.

It could be assumed that oxidative stress and inflammation of the nervous system are the culmination of changes in the periphery [54,55], which, due to the increased permeability of the blood–brain barrier, might lead to increased infiltration of peripheral material into the brain, thus constituting a potential pathogenetic factor of the disease. Presumably, it is pro-inflammatory cytokines that modulate mood behavior and cognition by reducing the level of monoamines in the brain, or promoting glutamate excitotoxicity and, thus, affect the neuronal plasticity of the brain [56].

The potential mechanisms of the development of schizophrenia presented in the literature for us are now the starting points in the construction of a statistical model based on objective laboratory data, linking peripheral and cerebral biochemical indicators related to common inflammatory mechanisms in schizophrenia. Such a model might facilitate the prediction of the severity of schizophrenia symptoms and the assessment of the development of early psychosis.

In addition to assessing the usefulness of selected biomarkers in determining the importance of clinical endophenotypes, the aim of the research was to create a new perspective on the use of laboratory parameters in the diagnosis of different clinical types of schizophrenia. This could form the basis for a personalized approach to the diagnosis of the disease, based on a reliable etiopathogenetic factor.

## 2. Materials and Methods

### 2.1. Study Participants

The research was approved by the Bioethics Committee of the Jagiellonian University (consent number: 1072.6120.152.2019 of 27 June 2019). The study included patients who gave informed written consent and additional consent was obtained in the case of legal guardians of participants under 18 years of age.

The study included patients with acute psychotic decompensation (*n* = 40; 18 women and 22 men; mean age 22.68 ± 7.39 years), admitted to the In-Patient Unit for Adults and In-Patient Unit for Adolescents at the Psychiatry Department of the University Hospital in Krakow. Recruitment for the study lasted from January 2018 to December 2019. For 70% of patients, it was the first psychotic episode. In the remaining cases, it was the subsequent psychotic decompensation resulting in hospitalization. All patients met the diagnostic criteria for schizophrenia (F20) according to the International Statistical Classification of Diseases and Health Problems, 10th edition (ICD-10) [7]. For relapsing patients, the diagnosis was confirmed during the initial assessment by the consensus of two psychiatrists with extensive experience in psychiatric assessment. For the first time, psychotic patients experiencing acute multiform psychotic disorders (F23) due to the ICD-10 diagnosis of schizophrenia was confirmed in a 3-month follow up. The severity of various psychotic symptoms was assessed using the Positive and Negative Syndrome Scale (PANSS) [57].

Exclusion criteria from the study were—inability to express informed consent, intellectual disability, hospitalization without consent or due to presence of severe cardiovascular diseases, abuse of psychoactive substances or tobacco smoking within three months prior to admission, affective symptoms, history of other disorders of the central nervous system, past head injuries with loss of consciousness, alcohol addiction, hyperactivity, or psychomotor agitation, which make it difficult to perform Magnetic Resonance Imaging (MRI).

Demographic and clinical data were collected from each patient, including duration of untreated psychosis (DUP), the course of the first episode, the number and length of hospitalizations, treatment, and its intervals.

During the first week of hospitalization, after medical stability was achieved, routine blood tests and questionnaire assessment were performed. MRI and MRS imaging examinations were performed during the first two weeks of hospitalization in patients who showed no changes in the outpatient status and were not undergoing pharmacotherapy, 8 h before each brain imaging.

In the constructed model of a number of variables (clinical, neuronal, biochemical, psychosocial), the potential dependencies of the brain activity indicators, peripheral parameters, and the clinical condition of patients were assessed.

### 2.2. Routinely Performed Laboratory Tests

After the subjects were qualified for the study, blood samples were collected during admission (1st day of the examination) using the Sarstedt closed system [58]. The patients fasted overnight and the collection was performed in the early morning hours. Samples showing bilirubinemia, hemolysis, lipemia, and turbidity were rejected.

Routine laboratory blood tests were performed on the day of sample collection and included a complete blood count (5-diff) and a manual smear, biochemical markers (ionogram—sodium [mmol/L], potassium [mmol/L], chlorides [mmol/L]; metabolic markers—glucose [mmol/L], lipidogram—cholesterol [mmol/L], HDL [mmol/L], LDL [mmol/L], triglycerides [mmol/L]; renal markers—creatinine [µmol/L], estimated Glomerular Filtration Rate test eGFR according to MORD [mL/min/1.73 m^2^]; inflammatory markers—C-Reactive Protein CRP [mg/L], cortisol [µg/dL], complement C3 [mg/dL], complement C4 [mg/dL]; thyroid markers—Thyroxine T4, Triiodothyronine T3, and Thyroid-Stimulating Hormone TSH), using the Sysmex XN-2000 automated analyzer (Kobe, Japan) for blood counts as well as the Cobas 6000 and Cobas 8000 biochemical analyzers (Roche Diagnostics, Mannheim, Germany) for the biochemical and hormonal parameters. Selection of the above-mentioned parameters is part of the obligatory routine management of patients admitted to the Psychiatry Department.

The excess of serum samples collected on the 1st day of the study was divided into aliquots and stored at –80 °C, and then used to evaluate the parameters of the oxidant–antioxidant balance (i.e., total antioxidant potential expressed as FRAP (ferric reducing ability of plasma))—paraoxonase 1 (PON-1, lipid peroxidation marker) and malondialdehyde (MDA).

### 2.3. Parameters of the Oxidant-Antioxidant Balance

#### 2.3.1. Rationale for the Assessment of the Efficiency of the Antioxidant System Expressed as FRAP in Schizophrenia

Activation of immune-inflammatory pathways is associated with oxidative stress and damage to lipids, nucleic acids, and proteins. Increased production of reactive oxygen species and weakening of the defense system associated with the action of antioxidants is the cause of oxidative stress (OxS) [59]. In turn, high antioxidant activity expressed as FRAP is associated with tardive dyskinesia, negative symptoms, neurological symptoms, dysfunction, and disturbances of total cholesterol metabolism in patients with schizophrenia [60,61].

#### 2.3.2. The Total Antioxidant Power Expressed as FRAP

The total antioxidant potential expressed as FRAP was determined by the spectrophotometric method of Benzie and Strain [62]. The spectrophotometric measurement provides information about the antioxidant capacity of plasma to counteract the effects of free oxygen radicals.

The reaction was performed in a 96-well microtiter plate on which 15 µL of the standard solution/test solution (serum) were placed, with water as the blank sample. Three hundred microliter of the working substrate solution (2,4,6-Tris(2-pyridyl)-s-triazine, TPTZ 0.01 mol/L, 0.02 mol/L FeCl_3_·6H_2_O, 0.3 mol/L pH = 3.6) was added into each well, the contents were mixed, and the plate was incubated at 37 °C for 10 min. The absorbance for each tested sample was determined at the wavelength λ_max_ = 593 nm. A standard curve was drawn (FeSO_4_·7H_2_O, 0.1–1, mmol/L) and a simple regression equation was determined from which the concentrations of the test samples were calculated; the results are expressed in mmol/mL.

#### 2.3.3. Rationale for the Assessment the Activity of Paraoxonase-1 (PON-1)

Human serum paraoxonase (PON-1) is an enzyme synthesized by the liver, which shows both paraoxonase and arylesterase activity to prevent peroxidation of low-density lipoproteins, i.e., LDL. PON-1 is involved in the transport of high-density lipoproteins [63]. The activity of PON-1 is altered in diseases where oxidative or nitrosative stress develops. Reduced PON-1 activity seems to be a key component of the oxidative and nitrosative processes that accompany schizophrenia [64].

The activity of paroxonase-1 (PON-1) in the plasma was determined according to the method described by Eckerson et al. [65], with the authors’ own modification. The absorbance of the resulting p-nitrophenol was recorded spectrophotometrically at λ_max_ = 405 nm at 25 °C, using a FLUOstar Omega microplate reader (BMG Labtech, Ortenberg, Germany). Plasma samples were mixed with a buffer containing 1.2 mM paraoxone in 50 mM glycine buffer, containing 1 mM CaCl_2_, pH = 10.5. Subsequently, samples were incubated for 15 min at 37 °C. In the next step, 20 µL of diluted plasma was measured, 200 µL of 1.2 mM paraoxone was added, and the absorbance value was monitored at 405 nm, every 15 s for 4 min, after prior gentle mixing.

The results were expressed in international units (U/L): PON-1 = OD/min × 11.4 = U/L.

#### 2.3.4. Rationale for the Assessment of the Lipid Peroxidation Product—MDA (Malondialdehyde)

Malondialdehyde (MDA) is one of the key indicators of oxidative stress and the end-product of lipid peroxidation. As a result of these processes, cell membranes are damaged and the amount of peroxygen polyunsaturated fatty acids in the brain is increased, which becomes a further easy target for an attack of reactive oxygen species (ROS), constituting one of the primary etiological mechanisms of schizophrenia [66,67].

The concentration of MDA was measured by the fluorimetric method described by Aust [68,69], in conjunction with the Gutteridge modification [70].

A working solution was prepared by dissolving the stock reagent, 2-Thiobarbituric acid, TBA/Trichloroacetic acid, TCA/Hydrochloric acid, HCl in water (on the assay day), thereby obtaining 3.5 mL TBA/TCA/HCl + 10.5 mL H_2_O + 0.21 mL BHT. The standard used was 1,1,3,3-tetramethoxypropane, which hydrolyzes in an acidic environment in a stoichiometric ratio to MDA. Hydrolysis was carried out in 0.05 mol/L hydrochloric acid at room temperature for 10 min, then standard solutions of 1,1,3,3-tetramethoxypropane were prepared in the range of 0.015–3.5 μmol/L. The working solution was prepared fresh daily from a solution containing TBA/TCA/HCl, by dissolution in water in a ratio of 1:3.

Test serum, blank, or reference samples were mixed with the working solution in the ratio—125 µL of the sample and 1000 µL of the working solution. The contents of the tubes were mixed for 10 s using a micro-shaker, and then heated in a boiling water bath for 15 min. The tubes were then immediately chilled on ice for 10 min, and 3 mL of butanol was added to each tube. The reaction mixtures were shaken for 30 s. After centrifugation for 10 min at 4000× *g* at room temperature, 250 µL of the organic layer were carefully transferred to the wells of a black 96-well plate. Fluorimetric measurements were made at an excitation wavelength (Ex) of 536 nm and an emission wavelength (Em) of 549 nm. The readings of the results were performed using the FLUOstar Omega spectrophotometer (BMG Labtech, Germany), after 10 min.

The clinical significance of the determination of the oxidant–antioxidant indicators selected by us was related to the evaluation of the action of antioxidant systems with the inflammatory theory of schizophrenia [71,72]. This links the role of immunological factors with the oxidant–antioxidant balance and the action of glutamatergic systems in the brain, departing from a theory focused solely on dopamine and emphasizing the role of inflammation and related mechanisms of action of new drugs [73,74].

Among the inflammatory parameters we selected, those that were routinely assessed, i.e., CRP, HDL, LDL, fibrinogen, and others are described above. In the case of redox parameters, the following were selected—paroxonase-1, which is similarly responsible for some anti-inflammatory and antioxidant properties, especially in the context of e.g., HDL [75]. The total antioxidant capacity of the plasma expressed as FRAP was selected as a clinical indicator of oxidative stress [76]. The choice of malondialdehyde was dictated by the assessment of the intensity of the lipid peroxidation process, as an indicator of oxidative stress [77].

### 2.4. Clinical Evaluation

Clinical evaluation of patients was based on a detailed psychiatric assessment. Demographic data were also collected. The psychiatric assessment included a clinical interview, the review of systems by a psychiatrist, neuro-cognitive tests, and a thorough analysis of the patient’s medical records and history. The severity of psychotic symptoms was assessed using the PANSS scale (Positive and Negative Syndrome Scale—PANSS positive, negative, general psychopathology, and PANSS total scores), which included the assessment of positive symptoms (e.g., delusions, hallucinations, excessive agitation, suspiciousness, hostility), negative symptoms (e.g., emotional withdrawal, poor communication, stereotypical thinking disorders, lack of spontaneity, and fluency in conversation), and general psychopathology [57]. The latter is an important addition to the assessment of basic positive and negative symptoms, providing information on the severity of schizophrenia, which constitutes a benchmark during the care of a psychotic patient.

### 2.5. Neuroimaging

MRI and MRS was performed using the 1,5T (General Electric Healthcare, Milwaukee, WI, USA) magnetic field induction MR system, with an 8-channel headcoil (receive only) in the supine position. The study was conducted with the use of a standard MR brain examination protocol, which included the following sequences: T2-weighted, FLAIR, Diffusion weighted imaging (DWI), and T1-weighted. Assessment of brain morphology was performed in order to exclude pathological or congenital lesions. Additional analysis of diffusion in the ACC region was performed using the Functool image analysis software (GE Healthcare; Chicago, IL, USA). For each subject, apparent diffusion coefficient maps were calculated. Region of interest (ROI) was placed in ACC, adjusted to the anatomical size of the ACC area, and it was approximately 2 cm^2^. The value of the diffusion signal and ADC coefficient was automatically calculated from each ROI, given as mean value and standard deviation. DWI sequence was performed in axial plane (slice thickness 5.0 mm, spacing 1.5 mm, TR 8000 ms, TE 98 ms, FOV24 cm, and matrix 128 × 128). The diffusion weighted imaging for 𝑏 = 0, 1500, s/mm^2^ was oriented in three directions.

Magnetic resonance spectroscopy (MRS) was performed using the single-voxel technique (SVS). The MRS spectra were acquired using the point-resolved spectroscopy sequence (PRESS Point- Resolved Spectroscopy Sequence). PRESS sequence utilizes one 900 and two 1800 radiofrequency pulses. For water suppression, the CHESS sequence (CHEmical shift Selective Imaging Sequence) was used with a frequency-selective 900 pulse to selectively excite the water signal, followed by a dephasing gradient. To obtain good quality spectra, automatic shimming was used. The magnetic resonance spectroscopy (MRS) acquisition parameters were—35 ms TE, 64 averages were acquired. In this study, the MRS signal was collected from the anterior cingulate cortex (ACC), parallel and superior to the dorsal anterior surface of the corpus callosum, and centered on the interhemispheric fissure. The volume of interest (VOI) was approximately 8 cm^3^. The size of VOI was adjusted to the anatomical size of the area the spectrum was collected from. The duration of the sequence was 2 min and 12 s.

During the spectroscopic examination, frequency adjustment of the transmitter and receiver was performed, as well as general correction of the magnetic field (schimming). Automatic schimming was routinely used during the prepscan. Schimming corrects the non-uniformity of the magnetic field. In the case of spectroscopy, the heterogeneity of the magnetic field is the widening of the peaks in the spectrum, the decrease in their amplitude and the signal-to-noise ratio, that hinders the attenuation of the water signal. Lack of this homogeneity leads to different Larmor precession values for protons coming from the same molecule. This causes the broadening of successive peaks in the spectrum, which is of particular importance for distinguishing between two closely adjacent peaks. The shim coils are responsible for maintaining the best homogeneity of the magnetic field. Homogeneity was measured through the width of the water peak at half its height. This is called half width FWHM (= full width at half maximum—a measure of the magnetic field homogeneity). The value FWHM and SNR (signal-to-noise ratio) were used to exclude the MR spectra of poor quality. The PRESS sequence was performed only when the FWHM was about 3. If the FWHM value was above 3, correction of the VOI alignment for spectroscopy was performed, and re-schimming was carried out. In exceptional cases, manual schimming was performed.

The spectroscopic analysis was performed with the SAGE 7.0 software (Plano, TX, USA).

Analysis was performed in the following steps—(1) zero filling (always to a power of 2), (2) Fourier transformation, (3) baseline correction (4) automatic phase correction, and (5) curve fitting, performed on the basis of a Gaussian shape to calculate the peak area.

In the analysis of the spectrum, the highest peaks at the 2.1 and 2.45 ppm locations were chosen as glutamatergic transmission metabolites—GLU (2.1 ppm) and GLN (2.45 ppm)—glutamate and glutamine.

Taking into account the parameters of the PRESS sequence, the prepscan used, and the subsequent steps of the spectrum analysis in SAGE, these peaks were marked separately.

### 2.6. Statistical Analysis

**Figure 2:**
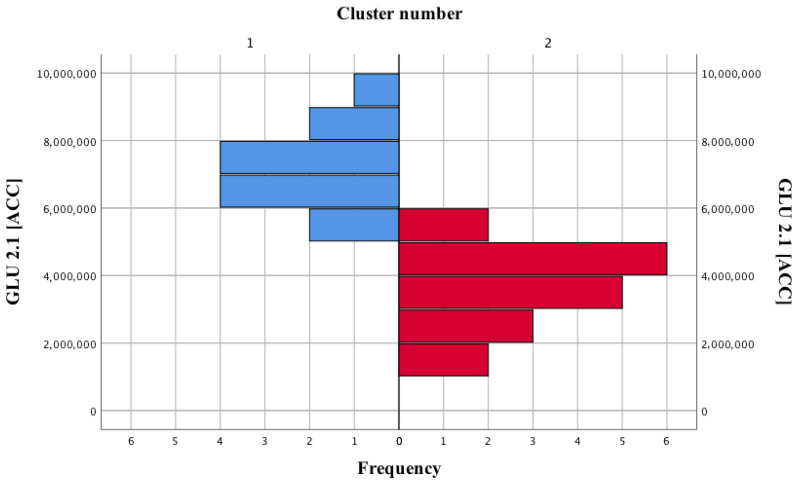
Separated clusters of subjects based on the GLU 2.1 (ACC) level, i.e., differing to the greatest possible extent in terms of the quality of life.

The statistical analysis was performed with the use of the IBM SPSS Statistics 25 package. Statistical, unsupervised (without a priori available knowledge) data analysis was used to determine the endophenotypes. An algorithm was used that divided the data into groups (clusters) so that each group was as homogeneous as possible, and at the same time the clusters were as different as possible. The results of division into clusters are presented in the form of charts (Figure 2). The following indicators were used independently for unsupervised k-means cluster analysis:

1. Brain metabolites, i.e.,—lipids (lip 0.9–1.0 ppm), lactates (lac 1.33 ppm), alanine (ala 1.48 mm), N-acetyl-aspartate (NAA 2.02 ppm), glutamate (glu 2.1 and 3.7 ppm), γ-aminobutyric acid (GABA 2.3 ppm), glutamine (gln 2.45 and 3.7 ppm), creatine (Cr 3.02 and 3.9 ppm), choline (Cho 3.22 ppm), glucose (glc 3.43 and 3.8 ppm), myo-inositol (mI 3.56 ppm), and glutathione (GSH 3.7 ppm), as well as the ratios of these metabolites in the frontal lobes—right, left, and ACC.
2. Biochemical parameters routinely determined as part of outpatient diagnostics, i.e.,: complete blood count (5-diff) and a manual smear, biochemical markers (ionogram—sodium [mmol/L], potassium [mmol/L], and chlorides [mmol/L]; metabolic markers—glucose [mmol/L], lipidogram—cholesterol [mmol/L], HDL [mmol/L], LDL [mmol/L], triglycerides [mmol/L]; renal markers—creatinine [µmol/L], eGFR according to MORD [mL/min/1.73 m^2^]: inflammatory markers—CRP [mg/L], cortisol [µg/dL], complement C3 [mg/dL], complement C4 [mg/dL]; thyroid markers—T4, T3, TSH);
3. Biochemical parameters reflecting the formation and action of reactive oxygen species and the related excess of oxidative stress, which is a response to the breakdown of the elements of antioxidant defense, i.e., MDA, FRAP, and PON-1. Their selection was dictated by the redox balance and inflammation of the nervous system in altered glutamatergic transmission, associated with disease symptoms [54]. Changes in the peripheral redox microcircuits presented in the study, due to the increased permeability of BBB, might lead to increased infiltration of peripheral material into the brain, and consequently might be a potential pathogenetic factor of the disease [51]. The brain’s susceptibility to stress, which leads to the overproduction of reactive forms of oxide, nitrogen, and sulfur, in conditions of impaired antioxidant defense, consequently causes damage to macromolecules, including extensive peroxidation of proteins, lipids, or nucleic acids, increased permeability of the blood–brain barrier and causes inflammation of the nervous system. Only when taken together, this can provide a reliable assessment of the centrally occurring changes in brain metabolism and morphology observed in mental disorders of a multifactorial nature [78].

The k-means cluster analysis allowed to distinguish 2 clusters of patients, based on the parameter GLU 2.1 in ACC. Using the Mann–Whitney U test, it was verified whether there were statistically significant differences between the selected clusters. The analysis of the Spearman correlation allowed to assess whether there is a statistically significant relationship between the analyzed variables. A *p*-value below 0.05 was considered to be statistically significant.

## 3. Results

### 3.1. Endophenotypes

About 700 cluster analyzes were carried out. From our data it was observed that patients with schizophrenia could be differentiated on the basis of presence of GLU 2.1 in ACC. In this way, two clusters (endophenotypes) were obtained, using the k-means cluster analysis—the main branches of the tree in Figure 2 marked with red and blue colors. These were characterized by the highest differences in terms of clinical parameters. The groups contained 41.9% and 58.1% subjects, respectively. The clusters separated in this way were equal, χ^2^ (1) = 0.81; *p* = 0.37.

Glutamate was also the only biochemical parameter that showed a statistically significant relationship with the clinical assessment of schizophrenics, i.e., the PANSS total scores, PANSS positive, and PANSS negative scale.

Based on the separated equal clusters, a comparison was made in terms of clinical evaluation indicators related to symptomatic diagnosis currently used in the diagnosis of schizophrenia (according to the PANSS positive, negative, general psychopathology, and PANSS total scores from the PANSS classification).

Descriptive statistics for GLU 2.1 (ACC) in the clusters of patients are presented in Table 1. Patients from cluster 1 achieved a significantly higher mean GLU 2.1 (ACC), compared to the second group of participants in the study.

### 3.2. Relationship of the Endophenotypes with the Clinical State of Patients with Schizophrenia

**Figure 3:**
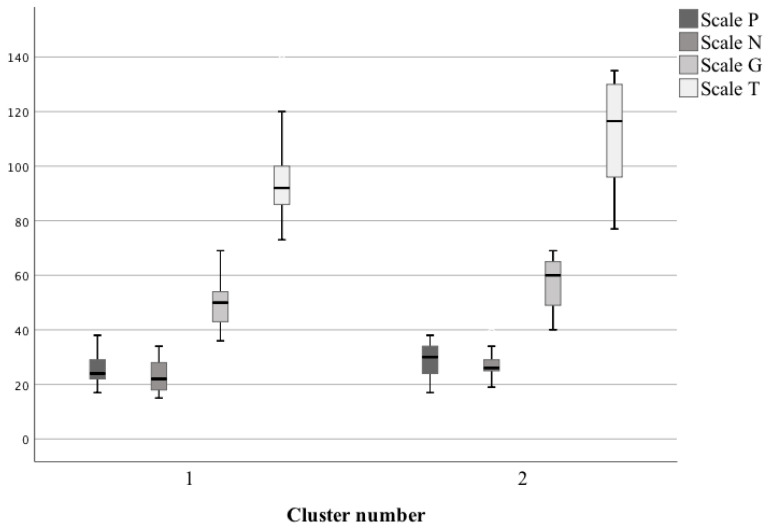
Scoring of the scales concerning the quality of life in selected clusters of patients.

Patients from the second cluster, i.e., with a reduced level of GLU 2.1 (ACC), had significantly higher scores on the N, G, and T scales, as compared to the first cluster. In the case of the P scale, the score was also higher, but the difference was not significant (Table 2, Figure 3).

**Figure 4:**
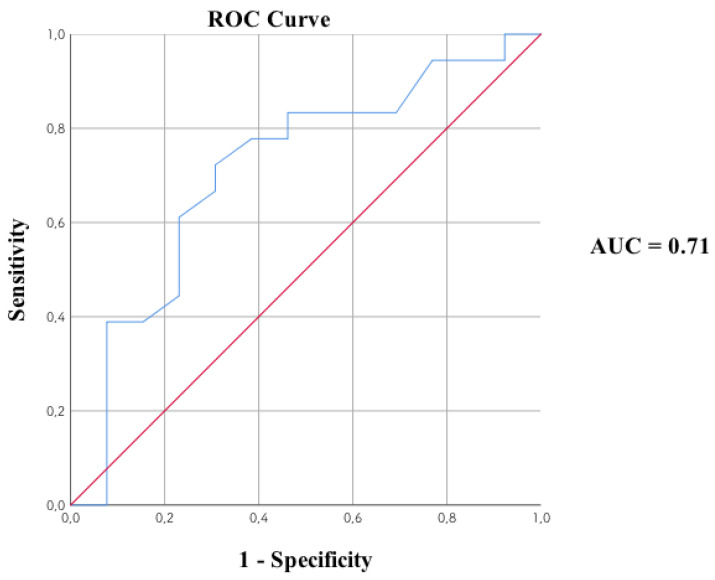
Predicting the membership of the second cluster on the basis of the T-scale scores. An ROC curve can be considered as the average value of the sensitivity for a test over all possible values of specificity or vice versa. A more general interpretation is that given the test results, the probability that for a randomly selected pair of patients with and without the disease/condition, the patient with the disease/condition has a result indicating greater suspicion. The area under the ROC (AUC) curve is bounded by the blue line and red baseline. The value of the AUC index is in the interval (0.1) delimited by two marked color lines. AUC, a high area under curve value limited by a blue line; ROC, a Receiver Operating Characteristic curve that includes all the possible decision thresholds from a diagnostic test result.

Additionally, a receiver operating characteristic (ROC) curve was prepared. The T scale allows us to significantly predict the belonging to the second group of people suffering from schizophrenia, i.e., with a reduced level of GLU 2.1 (ACC); AUC = 0.71 (Figure 4).

### 3.3. Relationship of the Endophenotypes with Routinely Determined Parameters

There was a difference in the level of neutrophils in the separate clusters. In research conducted by Bryll et al., it turned out that the biochemical parameters that showed the most numerous and strong correlations with the quality of life of the respondents were neutrophils and lymphocytes [21]. Both clusters obtained in our study differed in a significant way in terms of both analyzed variables (Table 3).

Patients from the second cluster, i.e., with a decreased GLU 2.1 (ACC) level, had a significantly higher percentage of neutrophils, as well as a lower percentage of lymphocytes, as compared to those from the first cluster. In our earlier studies, it turned out that the higher the percentage of neutrophils, the higher the scores of the individual scales [21]. A negative correlation was observed for the percentage of lymphocytes, which is presented in Table 4.

The relationships of GLU 2.1 (ACC) with the percentage of neutrophils (*r* = –0.49) and lymphocytes (*r* = 0.46) were statistically significant (*p* = 0.006 and *p* = 0.01, respectively). The presence of two stronger relationships GLU 2.1 (ACC) with the percentage of neutrophils and lymphocytes was observed: (1) neutrophil level, *r* = −0.49; *p* = 0.006; The higher the neutrophil count, the lower the GLU 2.1 (ACC) score; (2) lymphocyte level, *r* = 0.46; *p* = 0.01; The higher the lymphocyte count, the higher the GLU 2.1 (ACC) score.

### 3.4. Relationship of the Endophenotypes with Biochemical Parameters and Diffusion in the Anterior Cingulate Area

No other significant differences were observed between the biochemical parameters and diffusion in the anterior cingulate area in the clusters. The only significant difference was present in the level of GLU 2.1 (ACC) (Table 5).

In summary, two clusters of patients with schizophrenia were distinguished, based on the level of glutamatergic transmission metabolite in the anterior cingulate cortex (GLU 2.1 (ACC)), which differed in terms of symptoms, and also strongly differed in the percentage of neutrophils and lymphocytes (which, in turn, correlated with the quality of life).

No additional variables significantly differed between the two endophenotypes among those analyzed, i.e., parameters of oxidative stress (FRAP, MDA, and PON-1), diffusion (DWI), or biochemical indices.

## 4. Discussion

### 4.1. Endophenotypes

The medical world’s attention to the importance of subtypes in schizophrenia is not a new topic. It was Bleuler who independently pointed out the importance of the so-called ‘latent schizophrenia’, which emphasized not only the classic manifestation of the disease but also the different clinical pictures, from personality disturbances to affective psychosis. Interestingly, researchers believed that the symptoms of ‘latent schizophrenia’ interact with the underlying disease or the classic phenotype of schizophrenia, in which the concept of a latent trait is the basis of both glaring psychosis and other disorders described in the introduction. Bleuler, in his clinical observations, drew attention to the understanding of the definition of schizophrenia as a set of various disorders, defining them with the common term ‘group of schizophrenia’ [79].

The efforts of the last decade represent a breakthrough in the discovery of biomarkers that enhance symptomatic diagnosis and facilitate the prognosis of disease progression. Advances in the field of neuroimaging (NMR, PET, SPECT, fMRI, and MRS) and genetic techniques, opened the door to the characterization of clinical endophenotypes, which enriches the diagnosis and might constitute the basis for personalized medicine, related to the construction of appropriate statistical models to facilitate the diagnosis and treatment of neuropsychiatric diseases, including schizophrenia [46].

As a result of our research, two clusters of patients with an initial diagnosis of schizophrenia were defined in an unsupervised manner, based on the glutamatergic transmission metabolites level—Type I was characterized by a higher level of GLU 2.1, and Type II by its reduced level.

From the clinical point of view, due to the similar severity of positive symptoms but greater negative symptoms in the second endophenotype as compared to the first endophenotype, the second endophenotype might include patients with a less favorable course of schizophrenia, with more severe functional deterioration and requiring more intensive treatment. The obtained results might also suggest different risk factors for the development of schizophrenia in patients from both groups.

The glutamatergic hypothesis is one of the mechanisms explaining the observed results. One theory is that the insufficiency of the N-methyl-D-aspartate (NMDA) receptor leads to excessive release of glutamate in the frontal cortex, which might affect the release of dopamine and other neurotransmitters [80,81], which, in turn, leads to the expression of positive symptoms characteristic of schizophrenia [82]. It could also be explained by a higher density of D2 receptors in the striatum or an increased release of dopamine [83,84]. The results from the literature regarding the increased Gln/Glu ratio, explained by the abnormalities in the neuro-glial coupling, confirmed our results concerning the increased level of Glu in the endophenotype I [41]. These changes were more clearly marked in bipolar disorder, while in patients with acute psychotic episode, they only showed a tendency to be abnormal. These abnormalities appeared in both areas of the brain, suggesting that the lesions spread throughout the entire cortex. According to the hypothesis presented by Ongür et al., neuro-glial coupling abnormalities result in changes in the Gln/Glu ratio during acute episodes in the prefrontal cortex (ACC) and the parieto-occipital cortex (POC). Therefore, according to our previous results and reports of other authors, glutamatergic disorders might be associated with the period of illness or, sometimes, untreated psychosis [85,86].

The theories of glutamatergic transmission in schizophrenia suggest that the loss of NMDA receptors, especially in GABAergic interneurons, leads to disturbances in the excitatory–inhibitory balance and abnormalities of neuro-glial coupling in the prefrontal cortex. Oxidative stress is one of the initiating factors of schizophrenia, however, antioxidant systems or glutathione precursors (needed for the synthesis of glutamate and glutamine) might prevent changes in the synaptic transmission in pyramidal cells that result from the developmental NMDAR blockade and mitochondrial dysfunction with further generation of mitochondrial superoxides [87]. N-acetylcysteine (NAC) alleviates changes caused by the administration of ketamine, which confirms the participation of antioxidant systems in the changes generated by free radicals, with altered synaptic function [88]. Ketamine causes changes in the NMDAR function in prefrontal cortex interneurons, due to the increased generation of mitochondrial superoxides, mitigated by competition for the binding sites on NMDAR with NAC, which is considered to be a strong antioxidant [89,90]. Moreover, NAC administered at the last stage of maturation was also able to restore normal mitochondrial function and inhibition in pyramidal cells. This confirmed the role of peripherally located oxidant–antioxidant microcircuits on central macrosystems, and thus the self-control ability in preventing or restoring central changes. Moreover, this proves the validity of future schizophrenia treatment strategies targeting NMDA receptors and glutamatergic transmission.

The causes of the pathological sequelae arise from increases and dysregulation of mitochondrial ROS production and bioenergetic failure, which moves them from the periphery to the central nervous system. The mechanism of observed changes is connected with Nicotinamide Adenine Dinucleotide (NAD+) depletion, which raises ROS production on adenosine 5′-triphosphate (ATP) and NADPH generation and consistently decreases antioxidants and the glutamate signaling cascade in the anterior cingulate cortex is influenced [61].

Mitochondria, as the main source of energy production in the form of ATP and free radicals, play a key role in this regulation. The close relationship between the appearance of negative symptoms in schizophrenia and mitochondrial dysfunction, suggests that mitochondrial defects are crucial for disease development [91]. Disturbed glutamatergic transmission causes a reduction in GABA release and subsequent disinhibition of pyramidal cells with excessive release of glutamate [92] is visible in the group of patients with endophenotype I.

**Figure 5:**
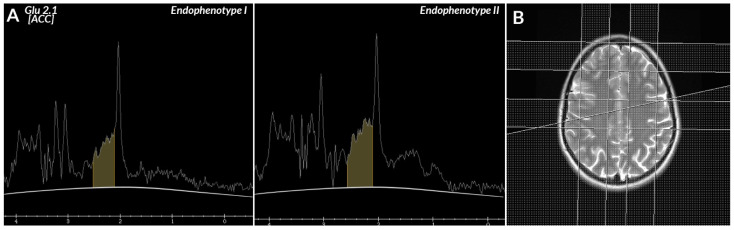
(**A**) On the MR spectrum, the yellow line is at the level of glutamatergic neurotransmission metabolites Glu, Gln (2.1–2.5 ppm) peak in endophenotype I, and the yellow line is at the level of Glu, Gln (2.1–2.5 ppm) peak in endophenotype II. (**B**) T2 weighted axial image, with the location of VOI in the ACC region.

Another mechanism explaining glutamate excitotoxicity in patients with endophenotype I might be a mechanism related to resistance to classic neuroleptics, which is in some way related to NMDA receptor failure, and results in a weakening of NMDA signaling, consequently leading to inhibition of stimulation due to insufficient activation of GABAergic neurons. As a result, weak negative feedback from GABAergic interneurons to pyramidal neurons increases glutamatergic neurotransmission, which then leads to glutamate excitotoxicity and develops resistance to drugs that act primarily on positive (not negative) symptoms (Figure 1 and Figure 5).

Disorders of glutamatergic neurotransmission might relate to another aspect of the pathophysiology of schizophrenia associated with the NMDA-R and its insufficiency, especially in the prefrontal cortex. NMDA-R mediates many neurobiological processes, including glutamatergic stimulation, brain structure development, and the induction of synaptic neuroplasticity. It was found that the NMDA-R deficiency in GABAergic neurons in the early postpartum period results in the development of schizophrenia. This type of receptor dysfunction is influenced by environmental factors. These include perinatal hypoxia or oxidative stress as important elements of NMDA-R deficiency in interneurons, which could be observed in the pilot studies conducted by our team [21].

Increased glutamatergic transmission levels in the ACC, as compared to healthy controls and patients responding to treatment were associated with the clinical endophenotypes identified in this study—endophenotype I in patients with schizophrenia who showed higher levels of glutamate (7,132,000.00 ± 1,167,008.62), in comparison to endophenotype II (3,574,438.89 ± 141,242.09) with a significantly reduced level of glutamate compared to endophenotype I. Results presented by the above-mentioned group of researchers suggest a relationship between the increased levels of glutamatergic neurometabolites and treatment-resistant schizophrenia (our results—7,132,000 ± 1,167,008.62 for *n* = 13 vs. the results of Demjaha et al. 8.87 ± 2.44, for *n* = 8 patients with schizophrenia in remission; or 10.32 ± 1.41 for *n* = 6 patients with treatment-resistant schizophrenia) [93]. In addition, the authors indicate the neurobiological foundations of resistance to treatment, suggesting their relationship with the action of glutamine in the ACC area [94].

In the work of Iwata et al., a similarly increased level of glutamate and glutamine in the ACC is explained by treatment-resistant schizophrenia. Consistent with the above-mentioned studies that showed higher levels of glutamine in ACC in patients with treatment-resistant schizophrenia, this study suggests that higher levels of ACC glutamatergic metabolites might be one of the common biological features of antipsychotic resistance in schizophrenic patients [95].

The phase of illness is not without significance, as the first episode of psychosis (FEP) and chronic schizophrenia constitute high-risk groups for which increased levels of glutamate and glutamine are found in the anterior and medial frontal cortex. Thus, higher levels of these metabolites occur in patients with more severe functional impairment, due to changes over time. The results of Dempster et al. showed no significant differences in the measurements of glutamatergic transmission metabolites in the ACC between patients who previously did not use drugs or used minimal treatment (FEP patients) and the control group [96]. This was consistent with our preliminary research, which was closer to explaining the importance of the glutamatergic theory in the pathophysiology of schizophrenia [21].

The levels of glutamate, glutamine, and other metabolites in the ACC showed an accelerated age-dependent decline [97]. Therefore, older people showed more pronounced decreases than the young. This additionally supported the clinical endophenotypes established in this study and their use in the group of younger people, who did not yet develop a visible difference from the healthy subjects [21] but showed likely changes before entering the psychotic phase, which could be considered to be a susceptibility to the disease [93,98].

The cluster II with a reduced glutamatergic transmission metabolites concentration selected in this study was explained in the works of Lutkenhoff et al. as a factor characterizing the obtained clinical endophenotype of patients with schizophrenia. This indicated the essential role of the reduced glutamate level in the quality of life in patients with schizophrenia [99]. This translated into clearly significant relationships between the decreased level of glutamate and negative, cognitive, and general symptoms obtained in this endophenotype, and a significantly smaller relationship with positive symptoms. This, in turn, confirmed the dopamine hypothesis of schizophrenia in eliminating the positive symptoms of the disease by classic neuroleptics, which left the negative symptoms unaffected.

The analysis of the relationships between the developed endophenotypes and the routinely measured parameters showed that neutrophils were most strongly associated with glutamate, while lymphocytes showed the opposite relationship. This confirmed the inflammatory hypothesis, which is often mentioned in the pathogenesis of schizophrenia. The positive relationship obtained in this study between cluster II (reduced glutamate level) and the level of neutrophils, as well as the negative relationship with lymphocytes, were confirmed in the study by Özdin and Böke [100]. The researchers observed that the ratio of neutrophils to lymphocytes (NLR) in patients during the exacerbation of the disease (the equivalent of our group with psychotic decompensation) was significantly higher, as compared to the control group. These studies confirmed the involvement of immunological and inflammatory mechanisms (outside the central one), emphasizing the role of the neutrophil–lymphocyte relationship with schizophrenia [101]. An additional explanation for the reduction of neutrophils in cluster I (increased glutamatergic transmission metabolites level) might be the response to the treatment of resistant schizophrenia, which could be interpreted as one of the side effects of polypharmacy. This would suggest additional phenotypic features of this endophenotype, such as greater exposure to neutropenia in the setting of polypharmacy and greater susceptibility to the effects of drug interactions [102].

In the group of patients with schizophrenia with a lower concentration of glutamatergic transmission metabolites (endophenotype II), we observed more severe clinical symptoms in all analyzed scales (P, N, G, T), and only the negative symptoms showed statistical significance. This might be associated with neurodegeneration or the consequences of chronic treatment usually linked with neutropenia [86,103]. However, this did not apply to our results characterizing patients with endophenotype II, for which a higher percentage of neutrophils than in the case of endophenotype I was observed. This rather indicated a substantial contribution of the inflammatory theory [71,72], which linked the role of immunological factors with the oxidant–antioxidant balance and the functioning of glutamatergic systems in the brain of schizophrenic patients [104].

Additional support for our results, emphasizing the role of the inflammatory theory related to altered glutamatergic transmission, was the lack of a relationship between local changes in the level of brain metabolites with the influence of previously used antipsychotic treatment, in a group of chronically schizophrenic patients [105].

An additional explanation for the reduced glutamatergic level observed in isolated subtype II and its statistically significant relationship with the symptoms (higher N, G, and T scores, compared to cluster I with elevated glutamatergic metabolites concentration) and the percentage of neutrophils, might be related to the release of inflammatory factors (such as chemokines, interleukins like MIP-2γ, or CXCL14). Their presence in the postsynaptic space reduced the expression of the glutamate-1 transporter on astrocytes and increased the sensitivity of neurons to glutamate excitotoxicity [106]. In addition, incorrect expression of glutamatergic transporters caused the accumulation of toxic concentrations of glutamatergic transmission metabolites and other free radical reaction products from the released neutrophils, which might also lead to inhibition of free diffusion (decrease in ADC, right frontal lobe, DEV) observed in the study in cluster II, as compared to cluster I. The degree of limitation of tissue diffusion was inversely proportional to cell density and cell membrane integrity. The higher the cell density, the number of intact cell membranes and, for example, the content of proteins/organelles (related to the infiltration of neutrophils), the more limited the free diffusion of water molecules, which could be seen in the study as an observed trend (without statistical significance). Consequently, changes in glutamatergic neurotransmission through reduced activity or expression of the glutamate transporter (GLT) might contribute to many observed neurological symptoms and the intensification of clinical symptoms. These results suggest that neutrophil-released inflammatory factors mediate the pathogenesis of CNS disorders associated with neutrophil infiltration into the brain and decreased GLT-1 activity. Overexpression of the toxic factors released by the infiltrating neutrophils, i.e., MIP-2γ, reduced the level of glutamate transporting proteins, i.e., GLT-1, resulting in glutamate excitotoxicity to neurons and astrocytes that removed excessive glutamate (observed increase in glutamate levels in cluster I).

### 4.2. Limitations

One of the limitations of the study was the use of a static model associated with a single measurement of the amount of metabolites (including glutamatergic transmission) in the 1H-MRS study, without the assessment of functional brain activation, allowing the assessment of changes in acquisition over time (1H-fMRS) [107]. This would give a more accurate assessment of the glutamatergic neurotransmission than the standard magnetic resonance spectroscopy [108].

The limitation of the methods used was conducting research related to MRS magnetic resonance spectroscopy on schizophrenia, at a relatively low-field strength of 1.5 T. A higher-field strength would provide a better signal-to-noise ratio (SNR) and thus a better spectral resolution, which in turn would allow for more precise quantification peaks of the studied metabolites of glutamatergic transmission. Field strengths, especially ≤3 T, have limited possibilities to quantify the overlapping glutamate and glutamine signals separately, which often makes their interpretation difficult. Multicenter cohort studies using high-field (7 T) MRS are planned, which would allow for improved detection of clear glutamate and glutamine signals in a group of patients at risk of developing schizophrenia and diseases from the differential diagnosis.

Additionally, the planned future research would determine the early response to the antipsychotic drugs, which seemed to determine the subsequent symptoms and functional outcomes of psychosis. Glutamate as an objective differential marker of patients with schizophrenia is a promising therapeutic target, especially for patients classified as endophenotype I, who are likely to show an insufficient response to classical treatment regimens.

We did not include the drugs used in the treatment of acute phase of schizophrenia in the analyses. These could affect both the glutamatergic activity of the brain and other investigated parameters, such as inflammatory markers, lipid profile, or blood morphology. There were several reasons for resigning from these analyses. The study was performed in the initial period of treatment of acute psychosis, accompanied by significant modifications of pharmacotherapy. Data on prior treatment were unreliable, due to the possibility of skipping prescription doses by patients, due to the increase in psychotic symptoms causing hospitalization. The study group was too small to analyze the effect of individual drugs or their groups. Difficulties with the use of drug equivalents in data mining were related to the use of both first- and second-generation drugs in the studied group that did not share pharmacokinetics, relations between dosage and effect, and affinity for various receptors, as well as the pro-inflammatory/anti-inflammatory effects [109].

The conducted study suggests that negative symptoms play an important role in young patients with an initial diagnosis of schizophrenia, especially in cluster II with low glutamatergic transmission metabolite levels in the MRS. The limitation of the obtained observations was the small sample size, however, due to the fact that our research provided strong evidence for linking the glutamatergic theory and a documented relationship with symptomatic diagnosis and inflammation (neutrophils/lymphocytes), we decided to present the results as the effect of research constituting a premise for further work on the endophenotypes identified in this research.

## 5. Conclusions

The multifactorial nature of schizophrenia and the lack of understanding of the role of biological factors in the etiopathogenesis of this disease, constitute the main limitation of the existence of an ideal biomarker. Thus, the introduction of new therapeutic approaches related to this disease is challenging.

Currently, there are no approved markers of risk stratification based on the multimodal bias of non-classical variants of schizophrenia and the proportion of rarer clinical endophenotypes with greater or lesser effect. We propose that the use of the glutamatergic marker(s) related to the functioning of the NMDA receptor in the anterior cingulate cortex could be used for diagnostic purposes in determining disease endophenotypes, which might lead to new diagnostic and therapeutic approaches and could provide more effective care for patients.

Personalized assessment based on the developed clinical endophenotypes of the disease seems to be highly justified in the stratification of young patients with schizophrenia. This should also be the main point of therapeutic activities related to the reduction of the severity of the axial symptoms of the disease. Negative symptoms of schizophrenia are often overlooked and difficult to treat, therefore, they are often accompanied by worse cognitive functioning or an increased risk of suicidal behavior. Glutamatergic transmission metabolites dysfunctions at the level of the anterior cingulate cortex appear first and spread to the prefrontal cortex [109], especially among adolescent patients with psychotic disorders, which is confirmed in the obtained endophenotypes (cluster II, which is most strongly associated with negative symptoms of the disease). Studies on a larger cohort might contribute to confirming the role of glutamatergic transmission in endophenotypes determination and might constitute an important premise for further research, especially among young people with an initial diagnosis of schizophrenia and negative symptoms.

Our study showed clear differences in the changes in the Glu level that objectively differentiate patients with schizophrenia (without a priori available knowledge), which is reflected in the different research hypotheses presented in the literature on the subject.

Further presentation of the results based on the influence of disease duration and phases (stable phase, decompensation, exacerbation, and symptomatic remission) on the level of Gln/Glu transmission seems justified.

## Figures and Tables

**Table 1:** Descriptive statistics for GLU 2.1 (ACC) in the selected clusters of patients with schizophrenia.

Cluster Number	GLU 2.1 (ACC) (Mean ± SD)
I	7,132,000 ± 1,167,008.62
II	3,574,438.89 ± 141,242.09
Statistical result	U = 0; *p* < 0.001

**Table 2:** Descriptive statistics on the quality of life of the two separate clusters of patients.

Cluster Number	P Scale (Mean ± SD)	N Scale (Mean ± SD)	G Scale (Mean ± SD)	T Scale (Mean ± SD)
I	25.85 ± 6.44	23.08 ± 6.03	50.35 ± 9.22	97.77 ± 19.46
II	28.94 ± 6.22	27.44 ± 4.71	57.22 ± 8.97	112.28 ± 19.15
Statistical result	U = 85.5 *p* = 0.21	U = 64 *p* = 0.03	U = 69 *p* = 0.049	U = 67.5 *p* = 0.046

**Table 3:** The level of neutrophils and lymphocytes in the selected clusters of patients with schizophrenia.

Cluster Number	Neutrophils [%] (Mean ± SD)	Lymphocytes [%] (Mean ± SD)
I	48.34 ± 7.67	38.95 ± 6.66
II	59.14 ± 8.78	29.35 ± 7.39
Statistical result	U = 44.5 *p* = 0.003	U = 45 *p* = 0.003

**Table 4:** Relationship between the percentage of neutrophils and lymphocytes in the studied group of people and their scores for individual scales.

Variable	P Scale	N Scale	G Scale	T Scale
Neutrophils [%]	0.46 *p* < 0.001	0.47 *p* < 0.001	0.48 *p* < 0.001	0.55 *p* < 0.001
Lymphocytes [%]	−0.4 *p* < 0.001	−0.45 *p* < 0.001	−0.46 *p* < 0.001	0.51 *p* < 0.001

**Table 5:** Descriptive statistics concerning the selected clusters of patients for biochemical parameters and diffusion in the anterior cingulate area.

Cluster Number	GLU 2.1 (ACC) (Mean ± SD)	FRAP (Mean ± SD)	MDA (Mean ± SD)	DWI, Frontal Lobes (AVG)		ADC, Right Frontal Lobe (DEV) (Mean ± SD)	PON-1 (Mean ± SD)
				Left (Mean ± SD)	Right (Mean ± SD)		
I	7,132,000 ± 1,167,008.62	0.36 ± 0.22	0.7 ± 0.16	333.69 ± 27.53	325.13 ± 33.09	0.00073 ± 0.0001	102.69 ± 3.79
II	3,574,438.89 ± 141,242.09	0.4 ± 0.23	0.78 ± 0.14	342.07 ± 41.02	343.07 ± 37.1	0.00069 ± 0.00064	101.08 ± 5.35
Statistical result	U = 0 *p* < 0.001	U = 105 *p* = 0.63	U = 114 *p* = 0.92	U = 85 *p* = 0.21	U = 99 *p* = 0.49	U = 94 *p* = 0.36	U = 101 *p* = 0.52

PON-1: Paraoxonase-1; MDA: malondialdehyde; FRAP: ferric reducing ability of plasma; DWI: diffusion-weighted imaging; AVG: average; ADC—apparent diffusion coefficient; and DEV: standard deviation.

## Data Availability

Not applicable.
